# Image quality and radiation dose of dual-source CT cardiac angiography using prospective ECG-triggering technique in pediatric patients with congenital heart disease

**DOI:** 10.1186/s13019-016-0460-9

**Published:** 2016-04-09

**Authors:** Ying Liu, Jian Li, Hongliang Zhao, Yan Jia, Jing Ren, Jian Xu, Yuewen Hao, Minwen Zheng

**Affiliations:** Department of Radiology, Xijing Hospital, Fourth Military Medical University, 169 West Changle Road, Xi’an, 710032 China; Siemens China Ltd., Shang Hai, China

**Keywords:** Dual-source CT, Congenital heart disease, Radiation dose

## Abstract

**Background:**

All studies involving use of ionizing radiation should be performed in accordance with the ALARA (As Low As Reasonably Achievable) principle, especially in children. In this study, the prospective ECG triggering technique with low voltage was used in dual-source computed tomography (DSCT) angiography to investigate if image quality with low radiation dose could be satisfactory in pediatric patients with congenital heart disease.

**Methods:**

Sixty pediatric patients with suspected congenital cardiovascular anomalies were enrolled prospectively in the study. They were randomly assigned to two groups for DSCT angiography. Group A were scanned by prospective ECG-triggering computed tomography angiography (CTA) with 80 kV tube voltage, while group B by used non-ECG-gated CTA with the same tube voltage. The anomaly accuracy was evaluated based on the surgical and/or conventional cardiac angiography findings. The overall image quality was assessed on a five-point scale. And the diagnostic accuracy and radiation dose was evaluated in both groups.

**Results:**

There were 127 cardiovascular anomalies in Group A and 108 in Group B. The mean subjective image quality and diagnostic accuracy between these two groups were significantly different (*P* = 0.007 and 0.011, respectively). The mean effective dose in Group A and Group B was 0.38 ± 0.13 mSv and 0.35 ± 0.17 mSv, respectively. But there was no significant difference between two groups (*P* = 0.197).

**Conclusions:**

The prospective ECG triggering technique in DSCT scan can offer better image quality and diagnostic accuracy with low radiation exposure in pediatric patients with congenital heart diseases. This technique has potential to become a new clinical routine in pediatric cardiac computed tomography (CT) imaging.

## Background

Cardiovascular computed tomography angiography (CTA) performed on multi-detector row scanners is an accurate method for noninvasive detection of congenital cardiovascular abnormalities [1–5]. Compared to retrospective ECG-gated computed tomography (CT), non-ECG-gated scanning technique is usually applied in CTA studies with low radiation dose in pediatric patients with congenital heart disease [6–8]. The non-ECG-gated technique has also been proved to be relatively more diagnostic accurate. However, it still remains difficult to show minor anatomic abnormalities because of motion artifacts according to the literature and also daily clinical experience [8, 9].

Sequential scanning technique with prospective ECG-triggering is a recent algorithm that could help to reduce the radiation exposure in cardiovascular CT [10, 11]. This technique only allows the ionized X-ray radiation to apply at a certain predefined period of the cardiac cycle rather than during the entire cycle as the retrospective ECG-gating technique. Therefore,prospective ECG-triggering can greatly lower radiation dose while ensure the diagnostic image quality of coronary artery [12–14]. Also, the clinical diagnostic demand of CT image quality is lower for congenital heart disease than for coronary artery disease [15]. Therefore, it is essential whether dual source CT (DSCT) cardiovascular angiography using prospective ECG-triggering can be performed in pediatric patients with congenital heart disease. The purpose of this study was to evaluate the image quality and diagnostic accuracy of DSCT cardiac angiography using low-voltage prospective ECG-triggering technique in pediatric patients with congenital heart disease in comparison with non-ECG-gated technique.

## Methods

### Patients

This prospective study was approved by the institutional review board and written informed consents were obtained from all patients’ guardians. The potential adverse effects of contrast medium injection and radiation exposure were explained to the parents by a cardiovascular radiologist.

A total of 60 consecutive pediatric patients younger than 5 years old were included in this study. All patients were referred to DSCT angiography for evaluation of suspected congenital cardiovascular anomalies for clinical reasons. Surgeries were performed in 47 patients, and 24 patients underwent conventional cardiac angiography (CCA). The final diagnosis of congenital heart disease was based on the surgical and/or conventional cardiac angiography findings. Patients with known hypersensitivity to iodine-containing contrast medium were excluded from this study. The mean age was 2.4 ± 2.1 years (range: 17 days ~ 5 years). The mean body weight was 7.2 ± 6.9 kg (range: 2.1 ~ 15.2 kg).

### DSCT protocol

All CT examinations were performed on a DSCT scanner (SOMATOM Definition, Siemens Medical Solutions, Forchheim, Germany). Before the exam, all patients were put under sedation by oral administration of chloral hydrate (50 ~ 75 mg/kg) according to the patient’s body weight and clinical condition. Abdominal bandage was used to reduce the respiratory artifacts during CT scan. All patients were randomly assigned to two groups for DSCT angiography (with 30 patients in each group). Patients in group A were scanned using prospective ECG-triggering. Patients in group B were examined by non-ECG-gated spiral CT scans. The scan parameters for both groups were listed in Table 1. All patients were scanned in a cranio-caudal direction from the lung apex to the liver dome to cover the entire lung parenchyma. Nonionic contrast medium Iopamidol (Iopamiro 370 mg I/mL, Bracco S.p.A.; Milan, Italy) was injected through a peripheral venous line by using a power injection. The injected volume was adjusted according to patients’ body weight: 2 ml/kg contrast medium followed by 20 ml of saline flush, both at a flow rate of 0.5 ~ 2.0 ml/s. For an optimum vascular opacification, a round region of interest (ROI) was placed in the left ventricle. The triggered threshold of ROI was set at 100 HU. Data acquisition was prospectively ECG-triggered, starting at 40 % of the R-R interval. The duration of the CT scans was 3.12 ~ 7.79 s.

**Table 1:** Characteristics of Patients and CT Scanning Parameters in Two Groups

Parameter	Group A	Group B
Number of children	30	30
Sex (male/female)	17/13	14/16
Mean age (Y)	2.6 ± 2.4	2.2 ± 1.8
Mean weight (Kg)	7.3 ± 7.1	7.1 ± 6.7
Tube voltage (kV)	80	80
Tube current time (mAs)	250	250
Collimation (mm)	0.6	0.6
Rotation time (sec)	0.33	0.50
Pitch	NA	1.0
Slice thickness (mm)	1.0	1.0
Reconstruction interval (mm)	0.8	0.8

### CT data postprocessing and image analysis

All reconstructed images were transferred to an external workstation (Leonardo; Siemens Medical Solutions, Forchheim, Germany). Multiplanar reformation (MPR), maximum intensity projection (MIP) and volume rendering technique (VRT) were used to visualize cardiovascular abnormalities. Diagnoses of cardiac abnormalities were proved by the surgical and/or conventional cardiac angiography. Two cardiovascular radiologists (with 7 and 10 years experience, respectively) subjectively interpreted image quality in consensus. A 5-point scale was used to grade the image quality (5 = excellent; 4 = good; 3 = fair; 2 = poor; 1 = not applicable) [16]. Images with score 3 or more were thought to be diagnostic.

The noise and signal-to-noise ratio (SNR) were measured in the ascending aorta and pulmonary trunk by one observe who was blinded to the subjective image quality scores. Image noise was defined as the standard deviation of the attenuation in a 50-mm^2^ circular ROI on the axial images with care take to avoid the walls [17]. SNR was calculated as the ratio of the attenuation value and the noise.

### Radiation dose estimations

The CT volume dose index (CTDI_vol_) and dose-length-product (DLP) values were recorded from the CT console displays for each scan. The effective dose was derived from the product of DLP and a conversion coefficient of 0.021 mSv/[mGy · cm] for the pediatric chest [18].

### Statistical analysis

Statistical analysis was performed on software SPSS (version 14.0, SPSS Inc.; Chicago, IL). Continuous variables were expressed as mean ± SD. The Student *t* test was used to analyze the difference of subjective image quality score, the image noise, SNR and radiation dose between the two groups. Diagnostic accuracy was compared by chi-square test between the two groups. Interobserver agreement in subjective image quality scoring was evaluated by kappa statistics. A к value of 0.61-0.80 was considered as good. A *P* value of less than 0.05 was considered to be statistically significant.

## Results

### Patient characteristics

Patient demographics were also shown in Table 1. No significant difference was found in patient age or weight between two groups (*P* = 0.375 and 0.631, respectively). The mean heart rates were 124.6 ± 17.3 beats per min (bpm) for group A and 127.9 ± 23.8 bpm for group B (*P* = 0.310).

### Cardiovascular diagnostic accuracy

Anatomical diagnoses of all 60 patients by surgical and/or CCA findings were listed in Table 2.

**Table 2:** Anatomical diagnoses of all 60 patients with prospective ECG-triggering DSCT angiography (Group A) and non-ECG-gated DSCT (Group B) with reference to surgical and/or CCA findings

Diagnosis	Group A /surgery and/or CCA	Group B /surgery and/or CCA
Tetralogy of Fallot	5/5	4/4
Aortic pulmonary septal defect	1/1	1/1
Anomalous pulmonary venous return	5/5	4/4
Aortic coarctation	6/6	8/8
Interrupted aortic arch	2/2	2/3
Pulmonary artery atresia with ventricle septal defect	3/3	2/2
Pulmonary artery atresia with intact ventricular septum	1/1	1/1
Anomalous origin of pulmonary artey	3/3	2/2
Transposition of the great arteries	2/2	3/3
Double outlet right ventricle	2/2	2/2
Total	30/30	29/30

A total of 106 separate cardiovascular abnormalities were confirmed by surgical and/or CCA findings in Group A, and 102 abnormalities in Group B (Table 3). For group A, prospective ECG-triggering DSCT angiography missed three abnormalities, including one small atrial septal defect and two pulmonary valve stenoses. However, non-ECG-gated DSCT missed ten abnormalities (Table 3) in group B. The total diagnostic accuracy of prospective ECG- triggering scan and non-ECG-gated scan was 97.2 % (103/106) and 90.2 % (92/102), respectively, which was significantly higher in group A than in group B (*P* = 0.011).

**Table 3:** Cardiovascular deformity findings with prospective ECG-triggering DSCT angiography (Group A) and non-ECG-gated DSCT (Group B) with reference to surgical and/or CCA findings

Abnormalities	Group A /surgery and/or CCA	Group B /surgery and/or CCA
Atrial septal defect	18/19	18/21
Ventricular septal defect	18/18	18/19
Patent ductus arteriosus	16/16	14/14
Aortic pulmonary septal defect	1/1	0/1
Anomalous pulmonary venous return	5/5	3/3
Aortic coarctation	6/6	8/8
Interrupted aortic arch	2/2	2/3
Coronary artery anomaly	2/2	0/1
Right aortic arch	0/0	2/2
Pulmonary valve stenosis	2/4	1/2
Pulmonary artery stenosis	11/11	9/9
Pulmonary artery atresia	4/4	2/3
Dilated pulmonary artery	11/11	9/9
Anomalous origin of pulmonary artey	3/3	2/2
Transposition of the great arteries	2/2	3/3
Double outlet right ventricle	2/2	1/2
Total	103/106	92/102

In the five patients with tetralogy of Fallot and scanned with prospective ECG- triggering acquisition, two coronary artery anomalies were found. Non-ECG-gated DSCT scan missed only one coronary artery anomaly in 4 patients with tetralogy of Fallot. These coronary artery findings were confirmed by surgery in all the patients with tetralogy of Fallot.

### Evaluation of image quality

**Fig. 1:**
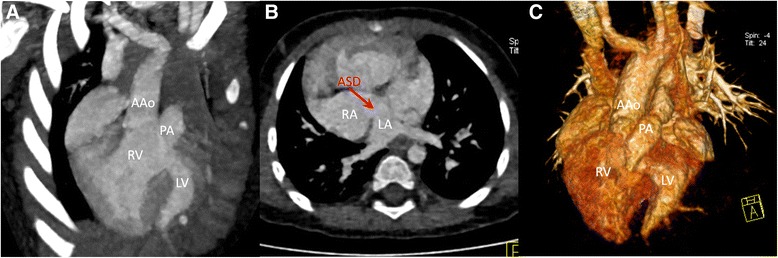
Dual-Source CT imaging with prospective ECG-triggering acquisition for a 15 month-old patient of double outlet right ventricle. Coronal MIP image (**a**) and coronal VR (**c**) show the ascending aorta (AAo) and the pulmonary artery (PA) both arise from the right ventricle (RV). Axial MPR image (**b**) shows this double outlet right ventricle is associated with an atrial septal defect (ASD). LV: left ventricle; RA: right atrium; LA: left atrium

**Fig. 2:**
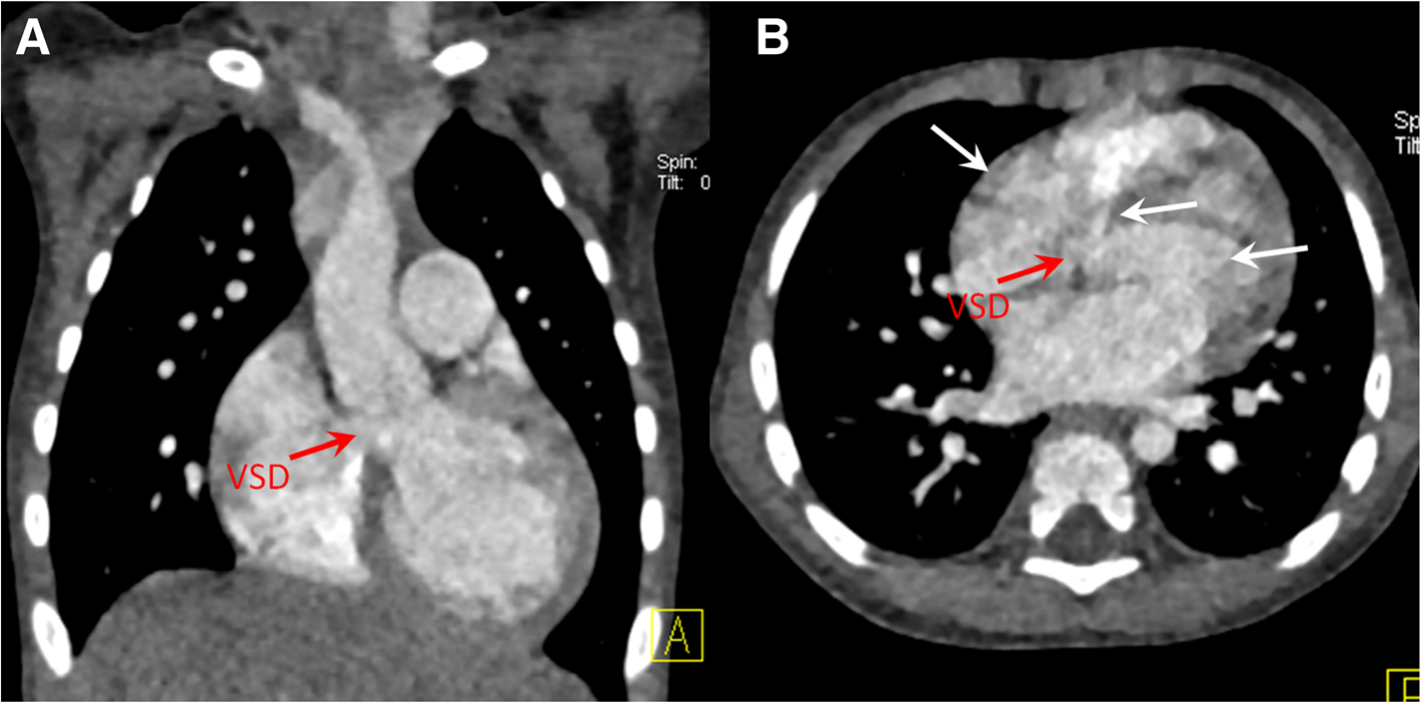
Dual-Source CT imaging with non-ECG-gated spiral acquisition for a 15 month-old patient of ventricular septal defect. Coronal MIP image (**a**) and axial MPR image (**b**) show a ventricular septal defect (VSD) with pulsation artifact (white arrows)

Interobserver agreement of the image quality was reached in all studies. The average subjective image quality scores of prospective ECG-triggering DSCT and non-ECG-gated DSCT were 4.4 ± 0.1 (range: 3–5) and 3.8 ± 0.3 (range: 2–5), respectively (*P* = 0.007) (Table 4). There was good agreement (к = 0.73) for overall image quality scoring between the two reviewers. The image quality with prospective ECG-triggering acquisition was found to be significantly better than with non-ECG-gated spiral acquisition (Figs. 1 and 2).

**Table 4:** Subjective and objective evaluation of image quality with prospective ECG-triggering DSCT (Group A) and non-ECG-gated DSCT (Group B)

	Group A	Group B	*P* value
Subjective image quality scores	4.4 ± 0.1	3.8 ± 0.3	0.007
Attenuation in the left atrium (HU)	409.2 ± 85.5	226.4 ± 141.6	0.003
Noise in the left atrium (HU)	40.0 ± 15.8	18.46 ± 5.6	0.004
SNR in the left atrium	11.7 ± 4.6	13.2 ± 8.9	0.638

*SNR,* signal to noise ratio

The image noise in the left atrium was significantly higher for the prospective ECG triggering CT scan group than that for the non-ECG-gated CT scan group. Also, it showed no significant difference of SNR between two groups (Table 4).

### Radiation dose

The mean CTDI_vol_ was 1.58 ± 0.65 mGy (range: 0.39-2.42) and 1.39 ± 0.71 mGy (range: 0.67-2.82) during prospective ECG- triggering acquisition and non-ECG-gated spiral acquisition, respectively. The mean DLP was 19.71 ± 10.56 mGy∙cm (range: 4–37) and 22.29 ± 13.00 mGy∙cm (range: 8–46), respectively, leads to a mean effective dose of 0.41 ± 0.22 mSv (range: 0.084-0.777) and 0.47 ± 0.27 mSv (range: 0.168-0.966), respectively for group A and group B. Although CTDI_vol_ was lower in group A, DLP and the effective radiation dose were higher in group A than that in group B, no significant difference was found between two groups (Table 5).

**Table 5:** Radiation dose parameters of prospective ECG-triggering DSCT (Group A) and non-ECG-gated DSCT (Group B)

	Group A	Group B	*P* value
CTDI_vol_ (mGy)	1.58 ± 0.65	1.39 ± 0.71	0.519
DLP (mGy · cm)	19.71 ± 10.56	22.29 ± 13.00	0.560
Effective radiation dose (mSv)	0.41 ± 0.22	0.47 ± 0.27	0.560

*CTDI*_*vol*_ *,* volume CT dose index; *DLP,* dose-length product

## Discussion

Any diagnostic test with ionizing radiation should be performed in accordance with the ALARA (As Low As Reasonably Achievable) principle, especially in children [19]. With the conventional non-ECG-gated DSCT scan, low dose radiation has been gained. However, for the infants and children who can’t hold their breath during CT scans, motion artifact has always been an issue and can severely impair the image quality and also lower the diagnostic accuracy. In order to reduce radiation dose while maintain the image quality, many dose-saving techniques have been developed, e.g., ECG-controlled dose modulation, tube voltage reduction and prospective-gating sequential scanning mode. Among these strategies, prospective ECG-triggering DSCT is considered one of the most useful methods to reduce radiation dose [20–24].

The reason of radiation dose reduction due to prospective triggering technique is that X-ray exposure only occurs during the selected cardiac phase rather than throughout the entire cardiac cycle (unless functional imaging is required [20]). Huang MP evaluated image quality and radiation dose at weight-based low-dose (80 kV and 60–120 mAs depending on weight) prospectively gated 256-slice MDCT angiography in 64 infants with congenital heart diseases [15]. Diagnostic images were achieved in all cases in Huang’s study, providing a comprehensive three-dimensional evaluation of the cardiac anatomy, including the coronary arteries. Jin KN and his colleagues compared the image quality and radiation dose of retrospective versus prospective ECG-gated dual-source CT imaging in 44 pediatric patients with congenital heart diseases [17]. They found that the image quality was better in the retrospective group than that in the prospective group. However, the mean estimated effective dose was much higher for the retrospective ECG-gated helical scan than that for the prospective non-helical scan, and the retrospective ECG-gated helical scan was definitely unfavorable choice for children. So far, it is known that prospective ECG triggering DSCT angiography can offer a low radiation dose choice. However, very few publications have demonstrated the difference of image quality between prospective ECG triggering and non-ECG-gated DSCT angiography with which low radiation dose can be obtained in both technique. In their study, image quality and the effective radiation dose using these two scan protocols with the same low tube voltage (80 kV) were investigated.

With a gantry rotation time of 330 ms, DSCT has a heart rate-independent temporal resolution of 83 ms with use of a single-segment reconstruction mode, which allows for cardiovascular angiography in patients with high heart rates, especially in infants and children [25]. Some publications [26, 27] reported that at heart rates higher than 85.5 bpm the best reconstruction time shifts to the end systolic phase. Therefore, in this study, 40 % of the R-R interval was used as the center of data acquisition window.

Results from this study showed that with low tube voltage (80 kV) there was no difference of the effective radiation dose between prospective ECG-triggering DSCT scan group (0.41 ± 0.22 mSv) and non-ECG-gated DSCT scan group (0.47 ± 0.27 mSv) (*P* = 0.560). However, the image quality score of prospective ECG-triggering DSCT scan group (4.4 ± 0.1) is significantly better than that of non-ECG-gated DSCT scan group (3.8 ± 0.3) (*P* = 0.007). Also, the image noise in the left atrium was significantly higher for the prospective ECG triggering CT scan group than that for the non-ECG-gated CT scan group. In accordance with the image quality, the diagnostic accuracy of prospective ECG-triggering DSCT scan group (97.2 % (103/106)) is also significantly better than that of non-ECG-gated DSCT scan group (90.2 % (92/102)) (*P* = 0.011).

There is a high incidence (11 %) of coronary artery abnormalities with congenital heart disease [28], especially with complex congenital heart disease. It is essential to evaluate coronary artery variations, because some variations can increase morbidity and even cause death during surgical correction [29, 30]. This study showed that prospective ECG-triggering DSCT scan could depict the coronary artery anomalies clearly, while motion artifact in non-ECG-gated DSCT scan group affected the image quality and led to the missed diagnosis of the only coronary artery anomaly.

### Study limitations

This study also has limitations. Firstly, it only included a relatively small group of patients. Future studies with larger patient cohort will be necessary. Secondly, heart beat variability was not considered. And also all the patients in this study showed regular sinus rhythm, however arrhythmia can affect the images massively.

## Conclusions

The prospective ECG triggering technique with low tube voltage in DSCT scan can reduce radiation dose and also maintain good image quality in pediatric patients with congenital heart diseases, as compared to a conventional non-ECG-gated DSCT angiography. This promising technique has a potential to become regular clinical method in pediatric CT scans.

## Abbreviations

CT: Computed tomography
CTA: Computed tomography angiography
DSCT: Dual-source CT
CCA: Conventional cardiac angiography
ROI: Region of interest
MPR: Multiplanar reconstruction
MIP: Maximum intensity projection
VRT: Volume rendering technique
SNR: Signal to noise ratio
CTDIvol: Volume CT dose index
DLP: Dose length product
